# Formula for a New Foam

**DOI:** 10.1289/ehp.112-a632

**Published:** 2004-08

**Authors:** Lance Frazer

By 2010, chlorofluorocarbons (CFCs) will be banned globally because of their adverse impact on the planet’s protective ozone layer. One industrial activity that has been significantly impacted by this ban is the manufacture of plastic foams—lightweight alternatives to solid plastic that are valued for their flexibility and ability to insulate, as well as their cushioning ability and (in marine applications) enhanced flotation.

Plastic foams are created by combining two chemicals that would otherwise form a solid plastic, or by melting an existing solid. A third substance, often a CFC, is then added as a “blowing agent.” This agent vaporizes at the reaction temperature, releasing gas bubbles into the molten plastic. “It’s much like soda,” explains Gordon Nelson, a professor of chemistry and dean of the Florida Institute of Technology College of Science and Liberal Arts. “Soda has carbon dioxide [CO_2_] trapped in it, but if you remove the pressure, you get fizz. With plastic, you get foam.” The resulting plastic foam can be exceptionally lightweight, given its size and application.

Today, the goal of the plastic foam industry is to make a material that remains lighter than solid plastic but has many of the same qualities of durability and flexible rigidity as the solid version, and to do so without having to rely on ozone-depleting gases. A team led by L. James Lee, a professor of chemical engineering at The Ohio State University, is pursuing one approach that relies on clay nanoparticles for strength and the green chemist’s old friend—supercritical CO_2_— to put the “foam” in plastic foam. Supercritical CO_2_, formed by putting CO_2_ gas under increasing temperature and pressure, has been used as an environmentally sound replacement for other toxic chemicals, including the solvents used in the manufacture of semiconductors [see “SCORR One for the Environment,” *EHP* 109:A382–A385 (2001)].

A number of researchers have been pursuing various types of nanoparticles as strengthening agents for plastic foams, while others have been looking at different substitutes for the banned CFCs; Lee, along with fellow associate chemical engineering professors Kurt W. Koelling and David L. Tomasko, decided to tackle both aspects at once. His ultimate goal—a lightweight, environmentally friendly plastic foam that may one day replace solid plastics in some applications.

## Better Bubbles

According to Lee, supercritical CO_2_ was a logical alternative to ozone-depleting CFCs. “Supercritical CO_2_ is a pretty benign substance,” he says. “And it’s neither difficult to make and use, nor is it expensive. Additionally, we’re not generating new carbon dioxide, merely using what’s available atmospherically, which lessens the impact still further.”

Lee says another advantage to supercritical CO_2_ is that under conditions necessary to reach supercritical status, the gas becomes a liquid, which mixes more easily with the molten plastic than a solid or gas. Lee says the optimal conditions arrived at in his experiments—temperatures just above 31°C and pressures of about 1,100 pounds per square inch—are easily achievable with current technologies. These results were reported in the 16 October 2003 issue of *Advanced Materials*.

And while most structural-grade plastic foams contain bubbles close to several hundred micrometers across, Lee’s process generates bubbles as small as 5 micrometers in diameter. According to Nelson, the bubbles must be small and uniform, or foams with less desirable qualities will result. “Smaller equals nicer consistency and better insulating properties,” he says. “Larger bubbles can alter the physical and thermal properties of the foam.”

There is one obstacle, however, according to Roland Loh, a global applications specialist for plastic foam manufacturer Owens Corning and principal investigator in the company’s search for environmentally benign nanocomposite foam products, and that is one particular property of CO_2_: “Carbon dioxide is a low-cost blowing agent, but its solubility is low, so if you want it diffused throughout the polymer, you need to keep it under pressure,” he says. “That will obviously raise the cost for industry.” Owens Corning is currently looking for a viable substitute for its blowing agent of choice, the CFC chlorodifluoroethane.

Lee admits that’s one of the aspects of the new technology still to be addressed. “The good thing about CFCs is that they dissolve better and diffuse out more slowly. Carbon dioxide is a small molecule, so it diffuses out more quickly, making it difficult to control density,” he explains. That’s one area where he and his colleagues believe the clay nanoparticles will help—by acting as a diffusion barrier, to slow the progress of the supercritical CO_2_ in diffusing out of the polymer.

## Mixing In the Particles

The addition of clay nanoparticles—the second aspect of Lee’s process—performs several critical functions in addition to improving the mechanical properties of the polymer. The clay also acts as a flame retardant and helps limit outgassing of potentially dangerous gases in the event the polymer does burn. These particles are added, less than 10% by volume, to the molten plastic.

According to Lee, the nanoparticles are derived from montmorillonite, an environmentally friendly, naturally occurring clay found in huge deposits in Europe and the United States. Clay naturally exists in a platelet-like structure, a structure maintained by particles even on the nanoscale, and this platelet-like nature can impart a number of desirable qualities. But the clay structure must first be modified before it can be added to the polymer.

In the 16 October 2003 *Advanced Materials* article, Lee explains: “In the natural state, [montmorillonite] platelets are held together by van der Waals forces and electrostatic forces to form crystallites (tactoids). Organic cationic surfactants, e.g., alkyl ammonium salts, are commonly used to modify the negatively charged clay surface through ion exchange, in order to improve wetting and lower the interfacial tension between the polymer and the clay that in turn enhances dispersion. Favorable interaction between the polymer matrix and clay surface and the resulting energy reduction are critical for the formation of exfoliated nanocomposites.”

Lee says one appealing aspect of clay as used in his work is that it forms a submicroscopic nucleation agent for the supercritical CO_2_, much the way impurities in a liquid or irregularities on the surface of a glass can trigger bubble formation. Reducing the pressure in the process that keeps the CO_2_ at a supercritical stage means the compound reverts to a gas, forming bubbles throughout the plastic. More nanoparticles within the molten plastic mean more bubbles will be formed throughout the plastic, and the large surface area of the nanoparticles provides much greater contact between the particles, the polymer matrix, and gases.

Clay, says Lee, is also an ideal strengthening agent because of its low cost. “Clay runs around two dollars per pound,” Lee says. “Other materials, like carbon nanofibers, would be even better, but a single-wall carbon nanotube can run five hundred dollars per gram.” Lee’s experiments have resulted in boards as strong as typical plastic foam, yet only two-thirds as thick. He’s experimenting with other strengthening materials, including aluminum and carbon.

Having thus far produced polystyrene and polymethyl methacrylate nanocomposite foams, Lee says his process can be used with any type of plastic, although certain polymers are more compatible. The process is better with polymers with high CO_2_ solubility, Lee explains. For example, poly-methyl methacrylate, used for optical fibers and as a shatterproof alternative to glass, has been a very good choice. Other good choices are polystyrene (used for applications including electric lawn and garden equipment and kitchen and bath accessories) and polyvinyl chloride (used for irrigation and other types of pipe). Polyolefins such as polyethylene (used in plastic bags, pipes, and packaging) and polypropylene (used for flexible food packaging) are more difficult. Additionally, the process will work as well, if not better, with non–petroleum-based plastics. “Clay is hydrophilic,” Lee explains, “so it doesn’t like to mix with oil. A non–petroleum-based plastic would be much easier for us to mix in the clay.”

## Feet of Clay?

Nelson says test results to date may not fully support the flame retardant aspects of clay nanoparticles. The presence of these particles will definitely reduce peak rates of heat release, he says, but in other tests, they haven’t shown they provide much benefit.

“Heat release is only one aspect of these industry tests,” Nelson says. “Does the material undergo sustained ignition? If so, then it’s not usable. . . . And the industry tests look at a broad range of things, like ignition, heat release, ease of extinguishment, and smoke and toxic gas formation.”

Nelson also notes that tests have shown variable flammability depending on whether the clay-augmented sample is oriented vertically or horizontally. In horizontal tests, clay comes to the surface and creates a barrier, which stops degradation and heat transfer. But in vertical tests, the clay barriers break, exposing new melting plastic—but not before effectively hindering the flow of the plastic just long enough to make it burn faster. “I think, from what I’ve seen,” he says, “that nanoclays may be useful in combination with traditional flame retardants, thus lowering the volume of flame retardant you need to use.”

Another concern, says Loh, is the concept of scalability. “The whole process of going from the lab to a commercial setting is always a challenge,” he says. “In the lab, you might be producing fifty grams of this stuff, while our pilot line might produce several hundreds pounds to a batch. The question is, how do you keep the same quality that you have in the lab? Labs use different equipment, and have different quality control aspects. The next stage for us in this project will be a pilot project, which we plan to have operational by year-end.”

Scale-up is always a challenge, agrees Lee. “We are successful in one scale-up with one company, and while I don’t have other data points at this time, it should, in theory, work in other materials and processes,” he adds. “The critical issue is to have good mixing and pressure/temperature control.”

Nelson thinks the issue comes down to application. He thinks it’s more likely that solid plastics will be replaced by better solids, and foams with better foams. He says, “Along with application comes the question of cost, which will always be the issue for industry. . . . What are the changes, what do they cost, and are they within reason? Also, you have to look at the qualities of the foam. Does it have the same initial properties? How does it age? These are the kinds of factors that industry will have to evaluate before adopting anything new.”

Despite the questions still outstanding, momentum for that adoption appears to be building. Lee’s team recently received a $2 million award from the Ohio state government’s Wright Capital Project Fund to introduce novel nanocomposites to industrial manufacturers. Besides Owens Corning, his work has also attracted the interest of Procter & Gamble. Only time will tell whether clay nanoparticles and supercritical CO_2_ are the answer for the plastic foam industry—but by 2010, at least, we should have an answer.

## Figures and Tables

**Figure:**
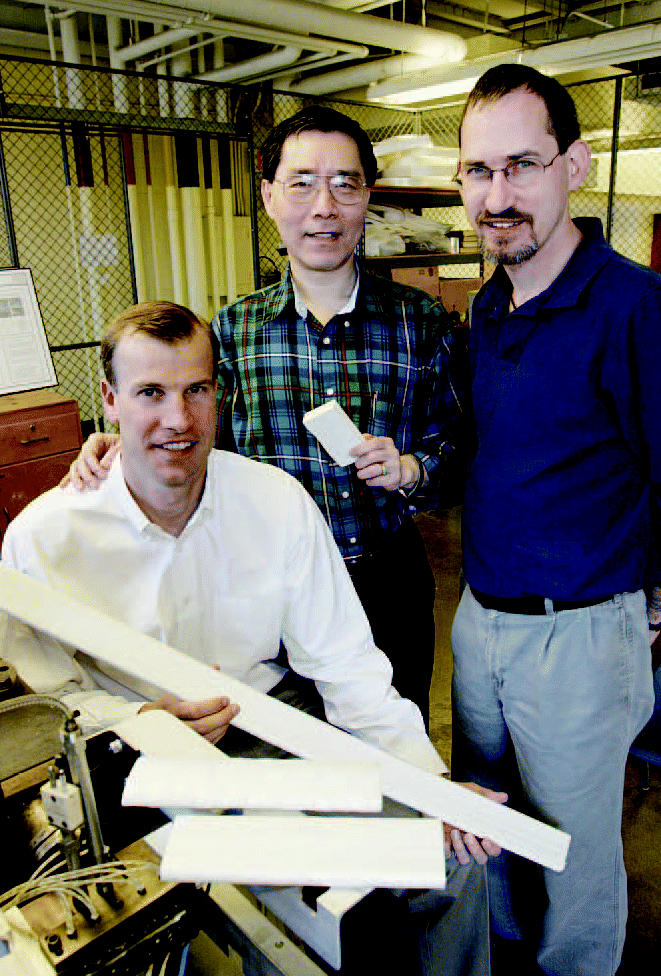
**A future in plastics?** (left to right) Kurt Koelling, L. James Lee, and David Tomasko with samples of the plastic foam materials they developed.

**Figure:**
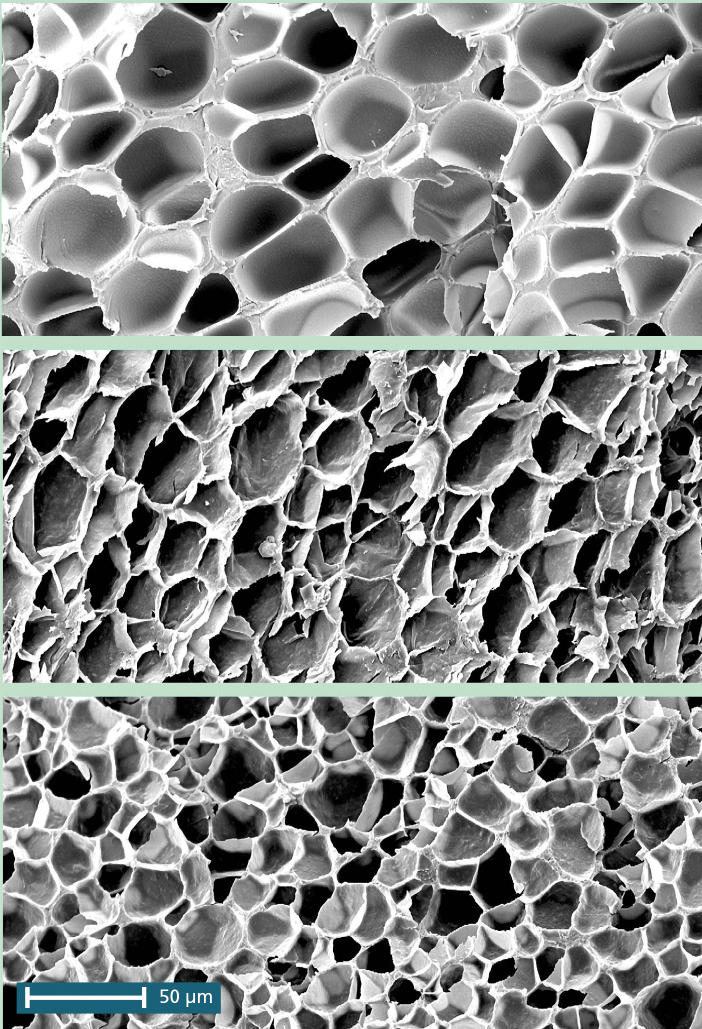
**Trans-foamation.** Micrographs allow comparison of cell size and density in polystyrene foams: (top) without clay, (middle) with intercalated clay, and (bottom) with exfoliated clay. Adding clay produces smaller, more uniform bubbles, resulting in a stronger plastic that insulates better.
